# Neurotrophic keratopathy due to dorsolateral medullary infarction (Wallenberg Syndrome): case report and literature review

**DOI:** 10.1186/s12883-014-0231-y

**Published:** 2014-12-04

**Authors:** Songdi Wu, Ningning Li, Feng Xia, Kastytis Sidlauskas, Xuemei Lin, Yihua Qian, Wei Gao, Qinlu Zhang

**Affiliations:** Department of Neurology, First Hospital of Xi’an, Xi’an, Shaanxi 710002 China; Division of Neuropathology, Department of Neurodegenerative Disease, Institute of Neurology, University College London (UCL), London, UK; Department of Neurology, Xijing Hospital, The Fourth Military Medical University, Xi’an, Shaanxi China; Department of Human Anatomy and Histology-Embryology, Xi’an Jiaotong University College of Medicine, Xi’an, China; Department of Ophthalmology, First Hospital of Xi’an, Xi’an, Shaanxi China; Xi’an GoldMag Nanobiotech Co. Ltd, 2 Zhang Ba Wu Road, Xi’an, Shaanxi China

**Keywords:** Dorsolateral medullary infarction, Neurotrophic keratopathy, Wallenberg syndrome

## Abstract

**Background:**

Dorsolateral medullary infarction (Wallenberg Syndrome) is rare in clinical practice; however, the subsequent corneal lesions are more uncommon. To our knowledge, only one such case was previously reported. We report a similar case with successful treatment and recovery, and analyse both cases to address the clinical features and outcomes of such syndrome.

**Case presentation:**

A 43-year-old male presented with neurotrophic keratopathy one month after sustaining dorsolateral medullary infarction. The patient underwent amniotic membrane transplantation twice. Two-year follow-up observation revealed changes in nerve fibers and epithelial cells of corneal by laser confocal microscopy.

**Conclusion:**

By studying both cases, we confirm that neurotrophic keratopathy could be as a delayed-onset complication of Wallenberg syndrome. The recognition that neurotrophic keratopathy can follow dorsolateral medullary infarction could prevent the clinical misdiagnosis.

## Background

The most common causes of neurotrophic keratopathy (NK) are herpesvirus infection, corneal surgery, diabetes and pathologic conditions that damage the peripheral fibers of the corneal nerve [1,2]. However, NK was rarely reported due to the dorsolateral medullary infarction (Wallenberg Syndrome). Here, we report NK as a delayed-onset symptom of dorsolateral medullary infarction and delineate its clinical features and treatment in conjunction with a previous case reported by Hipps WM et al. in 2004 [3].

## Case presentation

**Figure 1:**
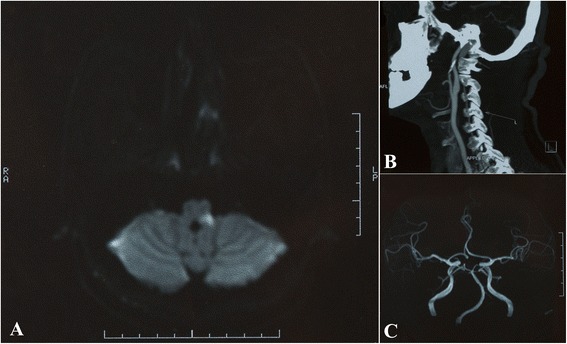
**Magnetic resonance imaging (MRI) and CT angiography findings. (A)** Diffusion-weighted magnetic resonance imaging showed region of hyperintensity in the left dorsolateral medulla. **(B)** CT angiography showed that the left vertebral artery became slender in the cervical part. **(C)** MR angiography showed a local blood flow disappearance of the left vertebral artery and basilar artery distortion.

A 43-year-old male visited the Department of Neurology, First Hospital of Xi’an in March 2011 and complained of sudden loss of movement in his left extremities accompanied with the symptoms of facial numbness and hearing impairment on the left, as well as dysarthria, cough, and vertigo. Neurological examination revealed left facial hypoalgesia, mild dysarthria, weak uplift of the left soft palate, and a slow gag reflex. Left upper limb hemiparesis with grade V^−^ power and left lower limb with grade IV power along with left unilateral hypoalgesia were noted. Babinski and Chaddock signs were positive on the left side. Diffusion-weighted MRI (DWI) revealed a region of hyperintensity in the left dorsolateral medulla (Figure 1A). MR angiography showed a local blood flow disappearance from the left vertebral artery as well as the distortion of the basilar artery. CT angiography showed that the left vertebral artery became slender in the cervical part (Figure 1B-C). The patient’s left hemiparesis slightly improved two weeks after antiplatelet therapy, but his left facial hypoalgesia and thermoanesthesia persisted. The patient suffered from non-controlled hypertension for three years, and had a drinking history of two years and smoking of one year. No history of diabetes, infections, surgeries or drug hypersensitivity was noted.

**Figure 2:**
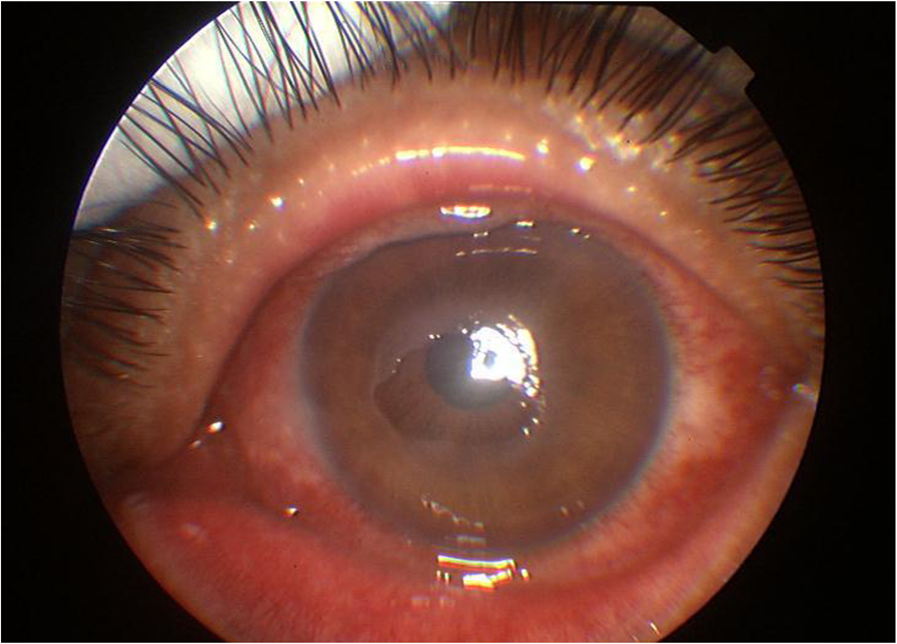
**Corneal findings.** An epithelial erosion and defect (sized 4 × 4 mm^2^) in the central corneal with conjunctival hyperemia was observed in the left eye.

One month after the stroke, the patient started to complain of dryness, foreign body sensation and blurred vision in his left eye. Three days later, corneal epithelial keratopathy occurred. The patient’s best-corrected visual acuity was 0.06 in the left eye and 1.0 in the right eye. An anterior segment slit-lamp examination showed conjunctival hyperemia and an epithelial defect (4 × 4 mm^2^) in the center of the cornea with mild stromal edema and entocorneal fold (Figure 2). Moreover, there was loss of corneal sensitivity in the left eye (tested by a gentle touch of the ocular surface with a wisp of cotton followed by observation of the blink reflex or by comparing the sensation with the other eye). No bacterial, viral or fungal growth was found in a culture of corneal scrapings. Due to the ineffectiveness of the antibiotic ointment (Levofloxacin Eye Gel, 3 day treatment) and nutrition therapy (Deproteinated Calf Serum Eye Gel, 3 day treatment), corneal debridement and amniotic membrane grafting (dry, stored, commercial amniotic membrane by JiXi RuiJi BioTechnology Co., Ltd) were performed in the left eye. During hospitalization, the weakness in the patient’s left extremities completely recovered and corneal epithelium healed, but facial hypalgesia was persistent. However, two weeks after discharge, corneal epithelial exfoliation recurred and became more severe. The second corneal debridement and amniotic membrane grafting were subsequently performed in the left eye alongside palliative supporting therapy.

**Figure 3:**
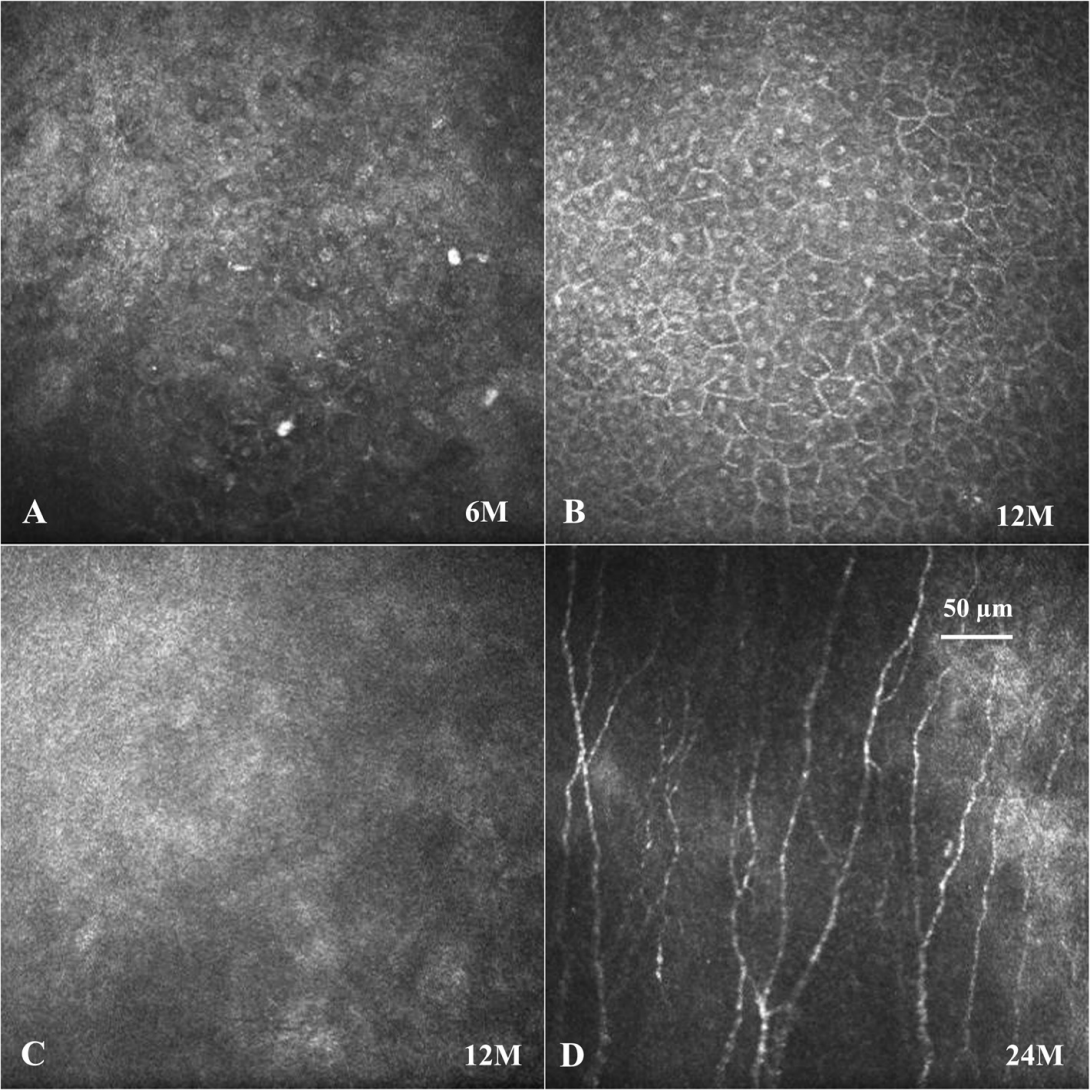
**Laser confocal microscopy examination findings.** Laser confocal microscopy examination showed that corneal epithelial cells was edematous, the boundary was not clear, and the connection was loose at post-operative month 6 **(A)**. The boundary of the epithelial cells of cornea were clearly recovered **(B)**, but the nerve fibers were still not found in the Bowman’s layer of cornea **(C)** at post-operative month 12. Nerve fiber and scar tissue can be found in the Bowman’s layer of cornea at post-operative month 24 **(D)**.

At post-operative month 6, laser confocal microscopy examination showed that the epithelial cells of the cornea were of irregular shape with no innervation observed in the Bowman’s layer of cornea (Figure 3A). Scar tissue and neovascularization was present in the superficial corneal stroma. The morphology of endothelial cells was normal. Twelve months post-operative outcome showed that the boundary of the corneal epithelial cells was clearly recovering (Figure 3B); however, nerve fibers were not found in the Bowman’s layer of cornea (Figure 3C). At post-operative month 24, nerve fibers and scar tissue were present in the Bowman’s layer of cornea (Figure 3D). Moreover, the eyesight of the left eye improved to 0.3. The corneal sensation of the left eye recovered to 70%.

## Discussion

It has been increasingly recognized that the corneal nerves play a key role in maintaining the anatomical integrity and functions of the cornea. Corneal nerve damage can lead to transient or chronic neurotrophic deficits with a decrease in the viability, metabolism, and rate of mitosis of the epithelial cells. This may result in epithelial intracellular swelling, loss of microvilli, and abnormal production of the basal lamina. As a consequence, symptoms of dry eye, corneal damage and infection develop known as neurotrophic keratopathy (NK) [1,2].

The vast majority of the corneal nerves are derived from the ophthalmic branch of the trigeminal nerve [4]. Therefore, NK can be induced by any damage to the corneal sensory nerve pathways [1,2]. The most common cause of NK is the peripheral corneal sensory nerve injury induced by viral infection, refractive surgery, chemical or thermal burns, ocular medication, and wearing contact lenses. Moreover, NK may develop from the damage of the ophthalmic branch of the trigeminal nerve as well as ciliary nerves due to tumor formation and surgery (such as cataract and retinal surgery or ophthalmic laser procedures). Other causes of NK include trigeminal ganglion lesions with herpetic viral infections and treatment of trigeminal neuralgia (e.g. microwave coagulation or ethanol injection in trigeminal ganglion), or preganglionic trigeminal nerve root injury (including acoustic neurinoma, schwannoma and aneurysma) and some systemic diseases which can decrease sensory nerve function or damage sensory fibres (e.g. diabetes and leprosy). As a result, brain stem injury could lead to NK, but relevant reports involving such injuries are rare [3].

A prerequisite for the diagnosis of NK is the decreased or lost corneal sensation [1,2]. Previously, Hipps et al. reported a 48-year-old male with persistent visual loss due to neurotrophic corneal ulceration after dorsolateral medullary infarction [3]. Reviewing Hipps’ and our cases we found that both responsible foci of infarction are located at the dorsolateral medulla as determined by MRI. Both patients developed serious and persistent ipsilateral facial hypalgesia and thermohypesthesia after the stroke. It is known that the lowest part of the spinal nuclei of the trigeminal nerve is related to the pain fibers from its ophthalmic branch containing corneal sensory nerves; therefore, damage to the lower part of the dorsolateral medulla may easily injure the corneal sensory nerve that comes from the lowest part of spinal trigeminal nuclei and result in interruption of the corneal sensory nerve pathway. Such injury may also cause secondary axis cord degeneration and myelin damage, finally leading to the occurrence of NK.

Therefore, we inferred that damage of the spinal trigeminal tract or nuclei, especially the lowest part, after medullary infarction is the root cause of the two cases of NK. The possible pathogenesis of the post-infarction NK may be the lost nutrient substance supplied by corneal nerve fibers, diminished protective blink reflexes induced by impaired corneal sensitivity, the desiccation of the corneal surface due to diminished lacrimal secretions, and abnormal epithelial cell metabolism with subsequent failure to resist the effects of trauma, drying, infection, etc. [1,2].

Further analysis of the two cases reveals the distinct features of dorsolateral medullary infarction induced NK compared to that caused by other lesions: 1) more serious and persistent ipsilateral facial hypalgesia and thermohypesthesia due to dorsolateral medullary infarction, 2) relatively delayed onset time of corneal damage (e.g. one to two months after dorsolateral medullary infarction), 3) more rapid disease progression once the corneal lesion appeared, 4) relatively serious clinical manifestations (e.g. corneal ulcers in the current case and corneal ulcers with infections in Hipps’ case), and 5) worse prognosis despite aggressive treatment for NK and dorsolateral medullary infarction. Whilst the patient reported by Hipps et al. [3] had permanent vision damage, we provided to our patient with relatively successful treatment, which may serve as a reference for other colleagues. Furthermore, laser confocal microscopy can helped to guide the degree of nerve damage and dynamic observation of corneal nerve regeneration, estimate the treatment, and the prognosis of the patients.

## Conclusion

Wallenberg’s syndrome was originally described in 1895 and typically presented with vertigo, dysarthria, ipsilateral ataxia, Horner syndrome, and decreased sensation on the contralateral body [5]. However, the combination of signs and symptoms varies according to the site of the lesion at dorsolateral medulla [6,7]. By studying both cases, we confirm that NK could be as a delayed-onset symptom of Wallenberg’s syndrome. Furthermore, we demonstrate that full recognition of this disease could prevent the clinical misdiagnosis.

## Consent

We obtained written informed consent from the patient for publication of this case report and any accompanying images. A copy of the written consent is available for review by the Editor-in-Chief of this journal.

## Abbreviation

NK: Neurotrophic keratopathy
